# A 1-year cross-sectional study on the predominance of influenza among hospitalized children in a tropical area, Kota Kinabalu, Sabah

**DOI:** 10.1186/s40101-022-00285-1

**Published:** 2022-04-02

**Authors:** Kai Joo Lim, Jecelyn Leaslie John, Syed Sharizman Syed Abdul Rahim, Richard Avoi, Mohd Rohaizat Hassan, Mohammad Saffree Jeffree, Mohd Yusof Ibrahim, Kamruddin Ahmed

**Affiliations:** 1 Sabah Women and Children’s Hospital, Ministry of Health Malaysia, 88996 Kota Kinabalu, Sabah Malaysia; 2 Department of Community and Family Medicine, Faculty of Medicine and Health Sciences, Universiti Malaysia Sabah, 88400 Kota Kinabalu, Sabah Malaysia; 3 Borneo Medical and Health Research Centre, Universiti Malaysia Sabah, 88400 Kota Kinabalu, Sabah Malaysia; 4 Department of Community Health, Faculty of Medicine, Universiti Kebangsaan Malaysia, 43600 Bangi, Selangor Malaysia; 5 Department of Pathology and Microbiology, Faculty of Medicine and Health Sciences, University Malaysia Sabah, Kota Kinabalu, Sabah Malaysia

**Keywords:** Influenza, Prevalence, Factors, Children, Sabah

## Abstract

**Background:**

Children are at higher risk of influenza virus infection, and it is difficult to diagnose. They are also responsible for the transmission of influenza because of their longer viral shedding compared to adults. In Malaysia, studies on influenza in children are scarce, and as a result, policy decisions cannot be formulated to control the infection. Hence, the objective of this study is to determine the prevalence and epidemiological characteristics of influenza among children with upper respiratory symptoms in the Sabah state of Malaysia.

**Methods:**

A cross-sectional study with a simple random sampling was conducted among children with upper respiratory symptoms in Sabah from 1 March 2019 to 29 February 2020. Patients admitted to a pediatric ward of Sabah Women and Children’s Hospital who presented with a fever >38 °C and cough within 48 h of admission were enrolled in this study. A nasopharyngeal swab was taken, and influenza was diagnosed by lateral flow test. Clinical features of influenza-positive children were compared with children whose results were negative.

**Results:**

A total of 323 nasopharyngeal samples were collected, and 66 (20.4%) of them were positive for influenza. Fifty-six (85%) were infected by influenza A whereas ten (15%) were by influenza B virus. Higher temperature (aOR 2.03, 95% CI 1.296–3.181), less activity (aOR 2.07, 95% CI 1.158–3.693), and seizure (aOR 4.2, 95% CI 1.614–10.978) on admission were significant risk factors associated with influenza in children. Meteorology parameters such as humidity and rainfall amount were statistically significant at 95% CI [1.133 (1.024–1.255)] and 95% CI [0.946 (0.907–0.986)].

**Conclusion:**

The prevalence of influenza was high among children with upper respiratory symptoms, and they were infected predominantly with the influenza A virus. Children presented with seizures, less activity, and fever were the significant risk factors for influenza. Influenza vaccination should be prioritized as preventive measures for children.

## Introduction

Globally, influenza epidemics cause 3 to 5 million severe illnesses and 290,000 to 650,000 deaths in a year [1]. In 2018, 10.1 million influenza virus-associated acute lower respiratory tract infections occurred among children under 5 years old globally [2]. Among them, 1.4 million and 0.4 million severe and very severe influenza respectively occurred in developing countries [2]. Despite this high number of mortality and morbidity among children in developing countries, studies on influenza in these countries are scarce. For example, in Malaysia, during the last 20 years, only four studies (excluding pandemic H1N1) have been done on influenza in children [1, 3–5]. Although in this country, respiratory infections rank second among the principal causes of infection, hospitalization (13.9%), and death (21.1%) [6]. Similar to other regional countries such as Thailand and Taiwan [7], in Malaysian children, the influenza virus is responsible for 6% of lower respiratory tract infections [8].

In Malaysia, the H1N1 pandemic in 2009 raised awareness among the public and healthcare staff regarding the importance and severity of influenza [9]. During that time, 1362 children were hospitalized with influenza-like illness (ILI); among them, 134 (9.8%) required intensive care and 51 (3.7%) died [10]. Furthermore, this infection became the spotlight again in 2019 when an 8-year-old child died of influenza in Sabah Women and Children Hospital, Kota Kinabalu, the capital of Sabah state in Malaysian Borneo. Kota Kinabalu is blessed with migratory aquatic birds, besides there are pig farms, which results in interactions between humans, pigs, and aquatic birds creating a potential situation for influenza transmission in the community. All these issues have persuaded us to understand the magnitude and epidemiology of influenza among the children of Sabah, which are needed for policy decisions on vaccination, treatment, and other control measures. Therefore, this study was performed to determine the prevalence and epidemiological characteristics of influenza among children with upper respiratory symptoms in a tertiary care hospital in Sabah.

## Methods

A cross-sectional study was conducted with a simple random sampling among the patients admitted to one of the three pediatric wards at Sabah Women and Children’s Hospital. This is a tertiary care referral hospital situated in Kota Kinabalu, the capital of Sabah state. Sabah is one of the thirteen states of Malaysia and is located in the northern part of Borneo Island. Kota Kinabalu has a population of 0.6 million people. Every day, 15 to 20 children are admitted to this hospital, and among them, 20 to 30% are from respiratory illnesses. This study was conducted from 1 March 2019 to 29 February 2020. Children with fever (>38 °C) and cough within 48 h of admission were enrolled randomly by lottery in this study. Children who had a history of nasal polyp or epistaxis were excluded from this study. The sample size was calculated based on formula hypothesis testing for 2 proportions. The age group < 6 months old, calculated with power = 0.8, *α* = 0.05, p1 and p2 of this study was 0.046 and 0.009, respectively [3], given a sample size of 302. With an add-on of a 10% drop rate from 302 samples, the sample size needed for this study is 332 with 28 samples per month for 1-year data collection.

The nasopharyngeal swabs were taken and tested at the bedside using a lateral flow test kit (Prolast Flu One®, Adtec Co., Ltd., Oita, Japan). This kit can detect the presence of influenza A and B viral nucleoprotein antigens in respiratory specimens and display the results as positive or negative. The sensitivity and specificity of the test kit are 91.4% and 95.0%, respectively. Written informed consent was obtained from the guardians of these enrolled children, and they were interviewed for information on sociodemographic characteristics, exposure factors, immunization status, and clinical features of children. All continuous variables were described using mean whereas categorical data were described as frequency (%). The data analysis was done using Statistical Package for Social Sciences version 22.0 (IBM Corporation, Armonk, NY, the USA). Univariate logistic regression was performed to compare sociodemographic characteristics, exposure factors, immunization status, and clinical features of patients with and without influenza. The results of univariate logistic regression, crude odds ratio (OR), p-value, and 95% confidence interval (CI) were determined for each variable. Multivariate logistic regression analysis was performed to predict factors associated with influenza. The final model was presented as adjusted OR (aOR) and 95% CI, Wald statistics, and p-value. The level of significance was set at a p-value less than 0.05.

Meteorological data with daily data points from March 2019 to February 2020 were obtained from the Malaysia Meteorological Department. A total of 52 data points by epidemiology weeks were analyzed. This data comprised 24 h of mean temperature (°C), 24 h of mean relative humidity (%), and rainfall amount (mm). This data represented the Kota Kinabalu station with the latitude of 5° 56′ N, the longitude of 116° 03′ E, and elevation of 2.1m with station no. 96471. Poisson regression analysis was used to analyze the relationship between meteorology parameters such as the mean temperature, humidity, and amount of rainfall with the number of children with influenza in this study. This analysis was done using the generalized linear models. Data was presented in time series distribution and Pearson chi-square was used to look at goodness-of-fit for time series data. 

## Results

### Prevalence of influenza

A total of 323 nasopharyngeal samples were collected from enrolled children (response rate >95%, only 2 parents rejected for consent), and their mean age was 3 years and 3 months old (ranged between 1 month old and 12 years old). Sixty-six (20.4%) of them were positive for influenza; among them, 56 (84.8%) and 10 (15.2%) were positive for influenza A and B virus, respectively. The mean age for influenza-positive cases was 3 years and 6 months old (ranging between 2 months old and 12 years old). Thirty-eight (57.6%) of the positive cases were in the age group of 1 to 5 years old followed by above 5 years old (28.8%) and less than 12 months old (13.6%) (Table 1). The male to female ratio of influenza-positive cases was 2.1:1.

**Table 1:** Comparison between influenza positive and negative cases based on different variables

Predictors	Influenza cases, *N* (%)		Total	Univariate logistic regression	
	Positive	Negative		Unadjusted OR (95% CI)	*p*-value
Nasopharyngeal samples	66 (20.4)	257 (79.6)	323		
Age					
< 12 months	9 (13.6)	68 (26.5)	77	1	
1–5 years old	38 (57.6)	124 (48.2)	162	2.315 (1.057–5.074)	0.036
> 5 years old	19 (28.8)	65 (25.3)	84	2.209 (0.932–5.234)	0.072
Gender					
Female	21 (31.8)	114 (44.4)	188	1	
Male	45 (68.2)	143 (55.6)	135	1.708 (0.963–3.031)	0.067
Race					
Malay	6 (9.1)	9 (3.5)	15	1	
Kadazan-Dusun	20 (30.3)	88 (34.2)	108	0.341 (0.109–1.067)	0.065
Bajau	14 (21.2)	76 (29.6)	90	0.276 (0.085–0.899)	0.033
Suluk	4 (6.1)	12 (4.7)	16	0.458 (0.147–1.430)	0.179
Others	22 (33.3)	72 (28.0)	94	0.500 (0.108–2.314)	0.375
Parent’s education level					
Tertiary	26 (39.4)	80 (31.1)	106	1	
Secondary	34 (51.5)	158 (61.5)	192	0.662 (0.372–1.179)	0.161
Primary	3 (4.5)	11 (4.3)	14	0.839 (0.217–3.241)	0.799
No formal education	3 (4.5)	8 (3.1)	11	1.154 (0.285–4.673)	0.841
District					
Kota Kinabalu	34 (51.5)	139 (54.1)	173	1	
Penampang	14 (21.2)	68 (26.5)	82	0.842 (0.424–1.673)	0.623
Others	18 (27.3)	50 (19.5)	68	1.472 (0.763–2.837)	0.249
House-Type					
Apartment / Flat	15 (22.7)	55 (21.4)	70	1	
Semi-detached house^a^	40 (60.6)	146 (56.8)	186	1.005 (0.514–1.962)	0.989
Others	11 (16.7)	56 (21.8)	67	0.720 (0.304–1.706)	0.456
Number of family members					
1–3	19 (28.8)	79 (30.7)	98	1	
4–5	21 (31.8)	110 (42.8)	131	0.794 (0.400–1.574)	0.508
6 and above	26 (39.4)	68 (26.5)	94	1.590 (0.810–3.121)	0.178
Contact history					
No	43 (65.2)	189 (73.5)	232	1	
Yes	23 (34.8)	68 (26.5)	91	1.487 (0.835–2.648)	0.178
Schooling					
No	51 (77.3)	184 (71.6)	235	1	
Yes	15 (22.7)	73 (28.4)	88	0.741 (0.392–1.401)	0.357
Pet at home					
No	31 (47.0)	154 (59.9)	185	1	
Yes	35 (53.0)	103 (40.1)	138	1.688 (0.980–2.908)	0.059
Immunization status					
Completed	64 (97.0)	255 (99.2)	319	1	
Not Completed	2 (3.0)	2 (0.8)	4	3.984 (0.551–28.830)	0.171
Influenza vaccination					
Yes	0 (0)	5 (1.9)	5	1	
No	66 (100)	252 (98.1)	318	423,100,930.2 (0.00)	0.999
Breast feeding					
Yes	38 (57.6)	155 (60.3)	193	1	
No	28 (42.4)	102 (39.7)	130	1.120 (0.647–1.938)	0.686
Underlying medical illness					
No	61 (92.4)	248 (96.5)	309	1	
Yes	5 (7.6)	9 (3.5)	14	2.259 (0.731–6.982)	0.157
Cough					
Yes	64 (97.0)	252 (98.1)	316	0.635 (0.120–3.348)	0.592
No	2 (3.0)	5 (1.9)	7	1	
Rhinitis					
Yes	62 (93.9)	251 (97.7)	313	0.371 (0.101–1.353)	0.133
No	4 (6.1)	6 (2.3)	10	1	
Seizure					
Yes	10 (15.2)	11 (4.3)	21	3.994 (1.617–9.864)	0.003
No	56 (84.8)	246 (95.7)	302	1	
Vomiting					
Yes	8 (12.1)	16 (6.2)	24	2.078 (0.848–5.089)	0.110
No	58 (87.9)	241 (93.8)	299	1	
Diarrhea					
Yes	13 (19.7)	54 (21.0)	67	0.922 (0.469–1.814)	0.814
No	53 (80.3)	203 (79.0)	256	1	
Child activity					
Not active	31 (47.0)	84 (32.7)	115	1.824 (1.053–3.159)	0.032
Active	35 (53.0)	173 (67.3)	208	1	
Appetite					
Absent	10 (15.2)	36 (14.0)	46	1.096 (0.513–2.343)	0.813
Present	56 (84.8)	221 (86.0)	277	1	
Sore throat					
Yes	7 (10.6)	18 (7.0)	25	1.575 (0.629–3.946)	0.332
No	59 (89.4)	239 (93.0)	298	1	
Temperature on admission					
38 °C and below	9 (13.6)	51 (19.8)	60	1	0.014
Above 38 °C	57 (86.4)	206 (80.2)	263	1.773 (1.125–2.795)	

^a^A semi-detached house shares one common wall with its neighboring home, whereby its layout will be a mirror image of the other

The influenza-positive patients were mainly from Kadazan-Dusun ethnicity (108; 30.3%), followed by Bajau (90; 21.2%) and other indigenous groups (94; 33.3%). Most of the parents of the influenza-positive cases attended secondary school (34; 51.5%) followed by those who attended tertiary education (26; 39.4%). Most of the participants (173; 53.5%) and influenza-positive cases (34; 51.5%) were from the Kota Kinabalu District.

When the place of residence was considered, we noticed that positive cases were mainly from patients who resided in semi-detached houses (40; 60.6%), followed by those from apartments (15; 22.7%). Besides that, positive cases were mainly from large families (26; 39.4%) and those who had pets (35; 53.0%). Most of the positive cases (43; 65.2%) had no contact history with family members who were ill and 51 (77.3%) of positive cases were not going to schoo.

Most respondents (97.0%) had completed compulsory free immunization under the National Immunization Program open for their age. Only five children (1.9%) had influenza vaccination, and none of them was positive for influenza virus. Among the positive cases, most of them were bbreastfed babies (57.6%) and had no underlying medical illnesses (92.4%).

Seizure, vomiting, diarrhea, and child’s activity on admission, appetite, sore throat, and temperature were the clinical features documented in this study. A child was considered as not active when the guardian described the child as malaise, not playing, or less active compared to normal days. One of the frequent symptoms other than respiratory symptoms was diarrhea (19.7%) followed by seizures (15.2%) and vomiting (12.1%). Among the positive cases, 53.0% were active, 84.8% had a good appetite, and 89.4% had no sore throat on admission. Univariate analysis between influenza-positive and influenza-negative cases showed statistically significant difference variables in the age group of 1 to 5 years old children, Bajau ethnicity, seizure, child’s activity, and temperature on admission.

### Predictive factors associated with influenza infection

The multivariate logistic regression model (Table 2) showed that influenza infection was associated with seizure within 48 h of admission (aOR 4.2, 95% CI 1.614–10.978), fever above 38 °C on admission (aOR 2.03, 95% CI 1.296–3.181), and children who are not active on admission (aOR 2.07, 95% CI 1.158–3.693). This model has a satisfactory fit, goodness-of-fit with a classification table of 79.3%, and *p* = 0.325 using the Hosmer-Lemeshow test.

**Table 2:** Multivariate logistic modelling on risk factors of influenza among hospitalized children

Risk factors	Positive, *n* (%)	aOR (95% CI)	*p*-value
Seizure			
No	56 (84.8)	1	
Yes	10 (15.2)	4.209 (1.614–10.978)	0.003
Temperature on admission			
38 °C and below	9 (13.6)	1	
Above 38 °C	57 (86.4)	2.030 (1.296–3.181)	0.002
Child’s activity			
Active	35 (53.0)	1	
Not active	31 (47.0)	2.068 (1.158–3.693)	0.014

*aOR* adjusted odds ratio, *CI* confidence interval

### Seasonality and meteorological factors

**Fig. 1:**
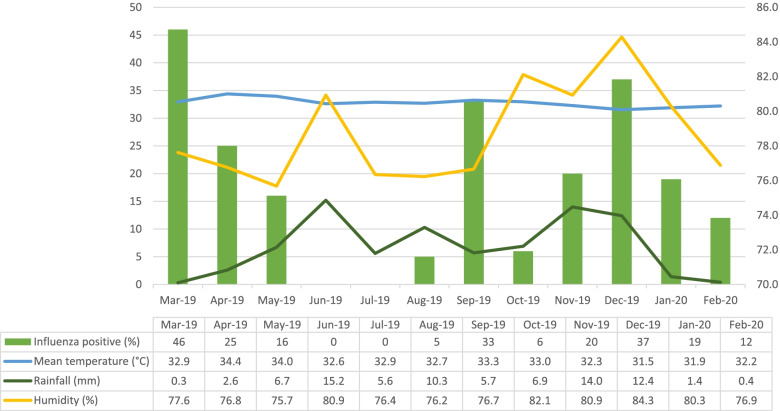
The monthly trend of influenza cases by humidity, rainfall, and temperature from March 2019 to February 2020

The mean temperature of Sabah is 28 °C with high relative humidity (mean of 78.6%) and heavy rainfall throughout the year. The monthly rate of influenza cases ranged from 0 to 46% (mean 18%). Three peaks (March, September, and December) of influenza were observed throughout the study period (Fig. 1).

The mean temperature, humidity, and amount of rainfall were 32.8 °C (ranged 31.0–34.8°C), 78.6% (ranged 70.4–85.5%), and 6.8mm (ranged 0–45.2mm), respectively.

Meteorology parameters such as humidity and rainfall amount were statistically significant in this study. Humidity is one of the factors that are statistically significant with 95% CI [1.133 (1.024–1.255)], whereby for every increase of 1% of 24 h mean relative humidity given the odd of 1.133 or 13% to have influenza infection among hospitalized children with upper respiratory symptoms (Table 3), rainfall amount is also statistically significant with 95% CI [0.946 (0.907–0.986)] whereby every increase of 1 mm of rainfall amount results in 5% reduction of influenza virus infection among hospitalized children with upper respiratory symptoms (Table 3). However, this data showed overdispersion and it was corrected with negative binomial regression analysis (Table 4). Since the confidence interval was almost the same as in Table 3, thus the result of Poisson regression analysis was selected.

**Table 3:** Poisson regression analysis on meteorology parameters and prevalence

Meteorology parameters	^a^Pearson chi-square	aOR (95% CI)	*p*-value
Mean temperature	2.146	0.899 (0.641–1.263)	0.540
Humidity		1.133 (1.024–1.255)	0.016
Rainfall		0.946 (0.907–0.986)	0.008

*aOR* adjusted odds ratio, *CI* confident interval

^a^Pearson chi-square showed 2.146 (>1), which indicated overdispersion data

*p*-value is based on likelihood ratio test

**Table 4:** Negative binomial regression analysis on meteorology parameters and prevalence

Meteorology parameters	^a^Pearson chi-square	aOR (95% CI)	*p*-value
Mean temperature	0.935	0.870 (0.491–1.540)	0.632
Humidity		1.146 (0.971–1.352)	0.107
Rainfall		0.941 (0.888–0.998)	0.042

*aOR* adjusted odds ratio, *CI* confident interval

^a^Pearson chi-square showed 0.935 (near 1), which indicated equidispersion data

*p*-value is based on likelihood ratio test

## Discussion

In this study, influenza infection among hospitalized children with upper respiratory symptoms was associated with seizures within 48 h of admission, fever above 38 °C on admission, and children who are not active on admission. Meteorological parameters such as humidity and rainfall amount are also significant in this study. Besides that, our finding is consistent with other studies which show that compared with younger children, older children are predominantly infected with influenza [11]. The exact reason for this is not clear; however, children older than 1 year are more likely to describe their illness and therefore receive early medical care [11]. Another reason which might contribute to an increased number of cases is that at this age, children have started attending nursery or school whereby influenza spreads easily. Older children are more susceptible to influenza probably because of the waning immunity that was conferred by breast milk in younger children and viral shedding is also longer compared to adults [12]. In our study, more than half of the children were breastfed babies. A breastfeeding mother can be encouraged to take the influenza vaccine during pregnancy which may provide protection for children up to 6 months old. The World Health Organization recommends influenza vaccination to prevent influenza-associated morbidity and mortality. Although influenza vaccination has a significant protective effect on children [13], in this study, only five children were vaccinated against influenza and they were all protected from influenza indicating the effectiveness of the influenza vaccine in this age group. Hence, we strongly think that if the influenza vaccine can be included in the National Immunization Program, this might increase the uptake of influenza vaccine among children and reduce the transmission of influenza among them. Because of limited data on the burden of influenza, the decision to introduce influenza vaccination under the National Immunization Program has not yet been possible in Malaysia. Therefore, comprehensive national surveillance is urgently needed to determine the burden of influenza-associated in this country. The influenza vaccine is not yet included in the National Immunization Program; however, it is available in private clinics by payment, and we think this is partly responsible for the low rate of influenza vaccination rate in Sabah.

However, determining the timing of vaccination will be challenging because the seasonal trend of influenza is more variable in tropical areas compared with temperate areas where a single peak incidence in winter leads to a specific timing of vaccination [14].

In our study, although influenza occurred all year-round as found in other tropical countries [3, 4, 9], however, there were three peaks of occurrences which are different from Singapore [15] and Vietnam [16] where two peaks were identified in November–December and February–March. Even though in the same South Asia region with a tropical climate, only one peak was observed in Indonesia during October–January [17] and in Thailand during June–September [7]. The monthly peaks observed in Sabah correspond to the mid-term school holidays in Malaysia which are usually 7–14 days break in March, September, and December. During school holidays, children travel to their hometowns with their parents. This activity may increase the intermingling of people, and hence, the transmission of the influenza virus. Strangely, similar to our study, influenza B peaked in late March among children in a temperate country South Korea [18]. However, consistent with studies in Malaysia [3] and abroad [19], we also detected that the prevalence of type B influenza was less than influenza A.

In tropical regions, influenza activity occurs year-round or with multiple outbreaks that happened during the rainy season [20]. A study done across temperate and tropical regions showed that two types of environmental conditions are associated with seasonal influenza, “cold-dry,” and “humid-rainy” [21]. It is postulated that in temperate regions, low humidity increased airborne transmission of influenza virus; however, in tropical regions, it is more likely transmitted by direct contact [20]. But in Hong Kong, the influenza activity was higher during their rainy seasons [20] which is similar to our study. Hence, the inclusion of meteorological parameters during surveillance may optimize public health decisions on vaccination strategy and healthcare resource preparedness [20, 22].

In this study, the number of boys was a little more than two times higher in getting influenza compared to girls as more boys were admitted with respiratory infections during our study which is consistent with others [23]. As expected, most children with influenza presented with respiratory symptoms; however, some children presented with seizures. The proportion of children in our study can be comparable to a study done in Italy which showed that 13.1% of children with influenza had central nervous system manifestation [24].

Seizure is one of the complications of influenza, and it occurs among two thirds of patients hospitalized for this viral infection with any neurologic complications at any age group [25]. Influenza A is one of the neurotropic viruses and is included as a causative organism to cause febrile seizures in Japan and China [26]. In influenza B virus infection, sometimes neurological manifestation can be the only symptom or may appear after respiratory symptoms [18]. Therefore, researchers are arguing that all children with acute neurological features during influenza season should be evaluated for influenza-associated central nervous system complications even if respiratory symptoms involvement is mild [24]. But in Malaysia, this is challenging as influenza is occurring year-round in contrast to that in temperate countries; therefore, further studies are needed to find out the relationship between neurological symptoms and influenza in tropical countries. In Malaysia, during the pandemic H1N1 in 2009, complications such as acute respiratory distress syndrome and encephalitis were presented among 10.4% of children admitted to the intensive care units [5]. Although in the present study, more children were presented with seizures but none of them were suffering from encephalitis. Children’s activity is another important factor associated with nearly half of our children with influenza. Children with less activity made up about 33.3–94% of children with influenza in different countries [11, 27].

The limitation of the current study is that only severe cases of influenza were included. Therefore, the extent of mild and moderate cases remains impalpable. Due to logistic issues, we were not able to perform genotyping of circulating influenza viruses. In the future, a state-wide study in different hospitals and genotyping of influenza virus is necessary.

## Conclusion

This study has provided evidence that influenza has a considerable impact on year-round hospitalization of children in tropical areas especially in Sabah. The prevalence of influenza was high among hospitalized children and influenza A was predominantly responsible for the infection. Children presented with seizures, fever, or less activity are risk factors for influenza and may be indicative of this infection. During this investigation, we noticed that influenza was not part of routine investigation for respiratory tract infections in children; therefore, we recommend that influenza rapid test kits to be made available at health care facilities especially in those that are dealing with children. Influenza vaccination should be prioritized for children to reduce the viral shedding period and minimize complications and mortality caused by this infection. In the future, a multi-center surveillance on the molecular characterization of influenza viruses and their distribution will reveal a clearer picture of this infection in Sabah.

## Data Availability

The datasets used and/or analyzed during the current study are available from KJL, Sabah Women and Children’s Hospital, Ministry of Health Malaysia, Kota Kinabalu, Sabah, Malaysia, on reasonable request.

## Abbreviations

ILI: Influenza-like illness
aOR: Adjusted odds ratio
CI: Confidence interval

## References

[1] Nair H, Brooks WA, Katz M, Roca A, Berkley JA, Madhi SA (2011). Global burden of respiratory infections due to seasonal influenza in young children: a systematic review and meta-analysis. Lancet.

[2] Wang X, Li Y, O’Brien KL, Madhi SA, Widdowson MA, Byass P (2020). Global burden of respiratory infections associated with seasonal influenza in children under 5 years in 2018: a systematic review and modelling study. Lancet Glob Health.

[3] Khor CS, Sam IC, Hooi PS, Quek KF, Chan YF (2012). Epidemiology and seasonality of respiratory viral infections in hospitalized children in Kuala Lumpur, Malaysia: a retrospective study of 27 years. BMC Pediatr.

[4] Sam I, Abdul-murad A, Karunakaran R, Rampal S, Chan Y, Marie A (2010). Clinical features of Malaysian children hospitalized with community-acquired seasonal influenza International Journal of Infectious Diseases Clinical features of Malaysian children hospitalized with community-acquired seasonal influenza. Int J Infect Dis.

[5] Koh MT, Eg KP, Loh SS (2016). Hospitalised Malaysian children with pandemic (H1N1) 2009 influenza: clinical characteristics, risk factors for severe disease and comparison with the 2002–2007 seasonal influenza. Singap Med J.

[6] MOH PDHIC (2019). Health facts 2019. Minist Heal Malaysia.

[7] Simmerman JM, Chittaganpitch M, Levy J, Chantra S, Maloney S, Uyeki T (2009). Incidence, seasonality and mortality associated with influenza pneumonia in Thailand: 2005–2008. PLoS One.

[8] Chan PWK, Goh AYT, Chua KB, Kharullah NS, Hooi PS (1999). Viral aetiology of lower respiratory tract infection in young Malaysian children. J Paediatr Child Health.

[9] Sam JI (2015). The burden of human influenza in Malaysia. Med J Malaysia.

[10] Muhammad Ismail HI (2011). Characteristics of children hospitalized for pandemic (H1N1) 2009, Malaysia. Emerg Infect Dis.

[11] Lin C, Chi H, Lin H, Chang L, Hou J, Huang C (2012). A scoring system for predicting results of influenza rapid test in children: a possible model facing overwhelming pandemic infection. J Microbiol Immunol Infect.

[12] Kondo H, Shobugawa Y, Hibino A, Yagami R, Dapat C, Okazaki M (2016). Influenza virus shedding in laninamivir-treated children upon returning to school. Tohoku J Exp Med.

[13] Suzuki T, Ono Y, Maeda H, Tsujimoto Y, Shobugawa Y, Dapat C (2014). Effectiveness of trivalent influenza vaccine among children in two consecutive seasons in a community in Japan. Tohoku J Exp Med.

[14] Laurie KL, Rockman S (2021). Which influenza viruses will emerge following the SARS-CoV-2 pandemic?. Influenza Other Respir Viruses.

[15] Chow A, Ma S, Ai EL, Suok KC (2006). Influenza-associated deaths in tropical Singapore. Emerg Infect Dis.

[16] Nguyen HLK, Saito R, Ngiem HK, Nishikawa M, Shobugawa Y, Nguyen DC (2007). Epidemiology of influenza in Hanoi, Vietnam, from 2001 to 2003. J Infect.

[17] Beckett CG, Kosasih H, Ma’roef C, Listiyaningsih E, Elyazar IRF, Wuryadi S (2004). Influenza surveillance in Indonesia: 1999-2003. Clin Infect Dis.

[18] Moon JH, Na JY, Kim JH, Yum MK, Oh JW, Kim CR (2013). Neurological and muscular manifestations associated with influenza B infection in children. Pediatr Neurol.

[19] Althaqafi A, Farahat F, Alsaedi A, Alshamrani M, Alsaeed MS (2021). Molecular detection of influenza A and B viruses in four consecutive influenza seasons 2015–16 to 2018–19 in a tertiary center in Western Saudi Arabia. J Epidemiol Glob Health.

[20] Chong KC, Lee TC, Bialasiewicz S, Chen J, Smith DW, Choy WSC (2020). Association between meteorological variations and activities of influenza A and B across different climate zones: a multi-region modelling analysis across the globe. J Infect..

[21] Tamerius JD, Shaman J, Alonso WJ, Bloom-Feshbach K, Uejio CK, Comrie A (2013). Environmental predictors of seasonal influenza epidemics across temperate and tropical climates. PLoS Pathog.

[22] Soebiyanto RP, Adimi F, Kiang RK (2010). Modeling and predicting seasonal influenza transmission in warm regions using climatological parameters. PLoS One.

[23] Weber MW, Mulholland EK, Greenwood BM (1998). Respiratory syncytial virus infection in tropical and developing countries. Trop Med Int Health.

[24] Mastrolia MV, Rubino C, Resti M, Trapani S, Galli L (2019). Characteristics and outcome of influenza- associated encephalopathy/encephalitis among children in a tertiary pediatric hospital in Italy , 2017 – 2019. BMC Infect Dis.

[25] Ekstrand JJ (2012). Neurologic complication of influenza. Semin Pediatr Neurol.

[26] Gordon Millichap J, Millichap JJ (2006). Role of viral infection in the etiology of febrile seizures. Pediatr Neurol.

[27] Monto AS, Gravenstein S, Elliott M, Colopy M, Schweinle J (2000). Clinical signs and symptoms predicting influenza infection. Arch Intern Med.

